# Novel Self-driven Microbial Nutrient Recovery Cell with Simultaneous Wastewater Purification

**DOI:** 10.1038/srep15744

**Published:** 2015-10-27

**Authors:** Xi Chen, Dongya Sun, Xiaoyuan Zhang, Peng Liang, Xia Huang

**Affiliations:** 1 State Key Joint Laboratory of Environment Simulation and Pollution Control, School of Environment, Tsinghua University, Beijing 100084, P.R.China

## Abstract

Conventional wastewater purification technologies consume large amounts of energy, while the abundant chemical energy and nutrient resources contained in sewage are wasted in such treatment processes. A microbial nutrient recovery cell (MNRC) has been developed to take advantage of the energy contained in wastewater, in order to simultaneously purify wastewater and recover nutrient ions. When wastewater was circulated between the anode and cathode chambers of the MNRC, the organics (COD) were removed by bacteria while ammonium and phosphate (NH_4_^+^-N and PO_4_^3−^-P) were recovered by the electrical field that was produced using *in situ* energy in the wastewater without additional energy input. The removal efficiencies from wastewater were >82% for COD, >96% for NH_4_^+^-N, and >64% for PO_4_^3−^-P in all the operational cycles. Simultaneously, the concentrations of NH_4_^+^ and PO_4_^3−^ in the recovery chamber increased to more than 1.5 and 2.2 times, respectively, compared with the initial concentrations in wastewater. The MNRC provides proof-of-concept as a sustainable, self-driven approach to efficient wastewater purification and nutrient recovery in a comprehensive bioelectrochemical system.

Global water shortages and resource crises caused by population explosion have become crucial concerns for the development of human society over the course of several decades. Researchers have focused on exploring new sources of water, energy, and nutrients; however, reusing water and recovering resources and energy from wastewater are even more important and sustainable, since wastewater contains abundant nutrients and chemical energy as well as providing a reusable water resource^1^. Conventional wastewater treatments have mainly focused on purification rather than resource recovery. The most commonly used process, aerobic/anoxic/aerobic (A^2^O) process, is mainly utilized for removal of COD and nutrients but not their recovery. Conventional treatment technologies therefore waste the nutrients and chemical energy contained in wastewater, while simultaneously consuming large amounts of electricity in the aeration process. Researchers have made great efforts to explore feasible technologies in attempts to recover resources and energy contained in wastewater. It was reported that adsorption using several kinds of sorbents could separate phosphate from wastewater^2^. Ion exchange, which acted as a reversible process, could be used to recover both ammonium and phosphate^3^,^4^. A common and practical approach is to simultaneously recover ammonium and phosphate as struvite from sludge digestion tanks where phosphorus is released to liquor from phosphorous enriched biosolids discharged from municipal wastewater treatment plants^5^,^6^,^7^. Enrichment of phosphorus from wastewater into activated sludge could be achieved by the group of polyphosphate-accumulation organisms (PAOs) through alternant anaerobic and aerobic conditions. However, this biological enrichment process is usually associated with energy consumption. Therefore, it would be significant if the nutrient could be cost-effectively and directly recovered from wastewater without additional energy input.

Bioelectrochemical systems (BESs) are a novel wastewater treatment technology that can oxidize substances in wastewater and simultaneously generate electricity or produce valuable compounds using bioelectrically active bacteria^8^,^9^,^10^,^11^,^12^,^13^. Typical BESs include microbial fuel cells (MFCs) that generate electricity^14^,^15^,^16^,^17^,^18^, microbial electrolysis cells (MECs) that produce hydrogen^19^,^20^,^21^, and microbial desalination cells (MDCs) that desalinate brackish water using pairs of cation and anion exchange membranes^22^,^23^,^24^,^25^,^26^. The emergence of MDC represents a new field in the use of electrical power derived from the energy in wastewater to realize direct migration of charged ions and thus water desalination. It has revealed the possibility that nutrients in ionic forms in wastewater could also be driven across membranes inside a BES. Several studies of nutrient removal via BES showed the potential for this application^27^. The BESs based on the MEC configuration which achieved nutrient removal with hydrogen production showed a great removal performance when an appropriate external voltage was applied^28^,^29^,^30^. A newly reported approach, termed R^2^-BES, attempts to recover nutrients from wastewater^31^. Ammoniums were migrated out of the wastewater by the driving force of bioelectricity while phosphates were exchanged with hydroxyls generated in a cathode reaction. The R^2^-BES could achieve the desired result with appropriate external voltages applied.

Inspired by the concept of MDC, a newly developed technology, termed a microbial nutrient recovery cell (MNRC) is presented here, for simultaneous efficient removal of COD and nutrients as well as *in situ* recovery of ammonium and phosphate ions, driven by the energy in wastewater. In the MNRC, a cation exchange membrane is positioned near the anode chamber, and an anion exchange membrane is positioned near the cathode chamber, thus forming a recovery chamber between the membranes. When wastewater is recirculated between the anode and cathode, the ammonium ions and phosphate ions are pushed into the recovery chamber from the anode and cathode sides, respectively, by the driving force of bioelectricity generated from wastewater. The removal performance of COD, ammonium, and phosphate were examined, and the recovery process was investigated in detail to reveal the extent of nutrient concentration and ion migration competition in the MNRC.

## Results

### MNRC operation and pollutant removal

**Figure 1:**
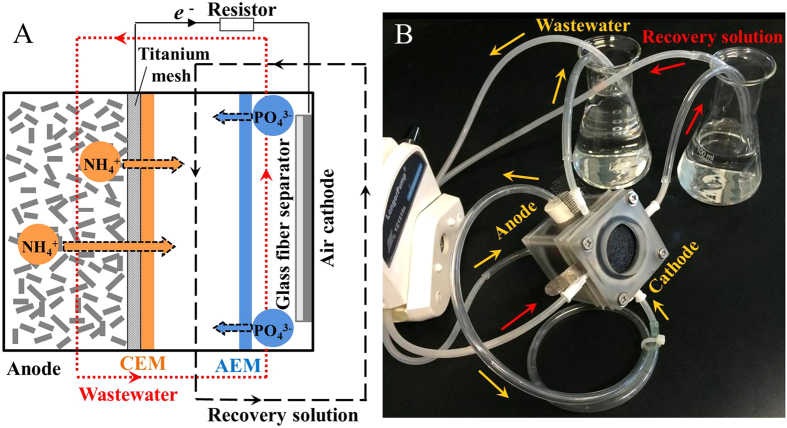
Schematic (A) and photograph (B) of microbial nutrient recovery cell (MNRC); AEM: anion exchange membrane; CEM: cation exchange membrane.

**Figure 2:**
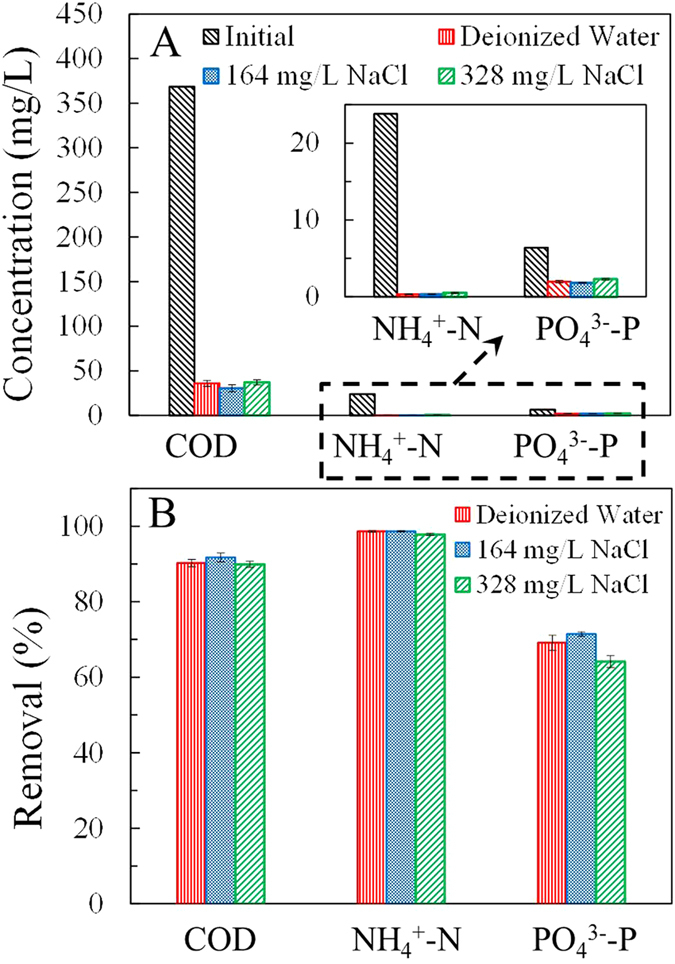
Concentrations in initial and treated synthetic wastewater (A) and removals (B) of COD, NH_4_^+^-N and PO_4_^3−^-P, using recovery solutions containing 0, 164, and 328 mg/L NaCl in a 24 h operational cycle.

The duplicated MNRC reactors each consisted of three chambers: an anode chamber, a recovery chamber, and a cathode chamber (Fig. 1). The synthetic wastewater, which acted as the electrolyte, was recirculated between the anode and cathode. The recovery solution, which received the nutrient ions migrated from the wastewater, was circulated individually. The MNRC effectively reduced the concentrations of COD, NH_4_^+^-N, and PO_4_^3−^-P in the synthetic wastewater over a 24 h operation cycle (Fig. 2A). The effluent contained COD < 37 mg/L, NH_4_^+^-N < 0.6 mg/L, and PO_4_^3−^-P < 2.2 mg/L when the MNRCs were operated with different concentrations of recovery solutions (0, 164, and 328 mg/L NaCl solution). Removals of these pollutants were >90% for COD, >97% for NH_4_^+^-N, and >64% for PO_4_^3−^-P in each experiment (Fig. 2B). These results confirm that synthetic wastewater could be well purified in the MNRC when the initial concentrations of recovery solutions varied within a certain range. However, removal performance still showed disparities among the three recovery solution concentrations. The highest removals of COD (92%), NH_4_^+^-N (99%), and PO_4_^3−^-P (71%) were simultaneously achieved with recovery solution containing 164 mg/L NaCl. In that experiment, COD was reduced from 369 mg/L to 30 mg/L, NH_4_^+^-N was reduced from 23.8 mg/L to 0.3 mg/L, and PO_4_^3−^-P was reduced from 6.4 mg/L to 1.8 mg/L. The differing removal results for different recovery solution concentrations might be caused by a balance between the MNRC internal resistance and ion diffusions in the recovery chamber. When the concentration of the recovery solution was increased, the conductivity was higher, leading to lower internal resistance and higher current production. However, with more concentrated recovery solution, the diffusion of Na^+^ and Cl^−^ from recovery chamber to electrode chambers would be reinforced, resulting in an opposed current flow and thus a detrimental effect on the overall current production. Therefore, the concentration of recovery solution could be optimized to obtain the maximum current, which would lead to optimal contaminant removal efficiency. The concentrations of Na^+^ and Cl^−^ in the 164 mg/L NaCl recovery solution were similar to those in the synthetic wastewater, and thus the undesired diffusion of ions could be minimized and the highest COD, NH_4_^+^-N, and PO_4_^3−^-P removals were achieved.

### Concentrating NH_4_^+^-N and PO_4_^3−^-P in the recovery chamber

**Figure 3:**
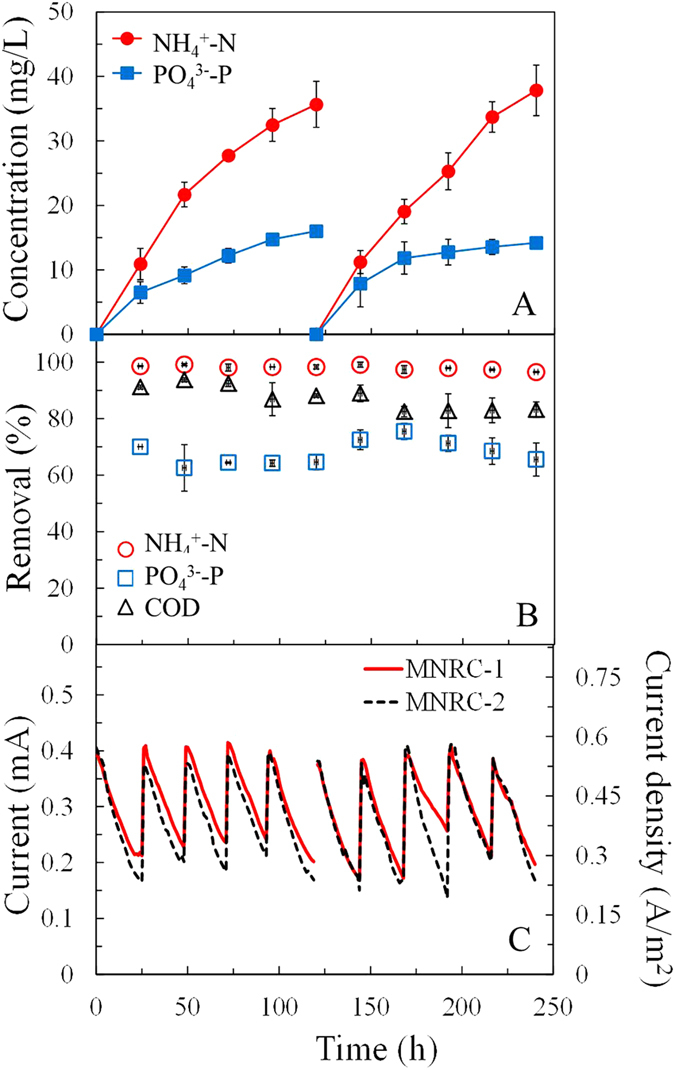
Concentrations of NH_4_^+^-N and PO_4_^3−^-P in the recovery solution (A), removals of COD, NH_4_^+^-N, and PO_4_^3−^-P in wastewater (B), and current generation of the duplicated MNRCs (refer to as MNRC-1 and MNRC-2) (C) in two repeated concentration tests.

NH_4_^+^-N and PO_4_^3−^-P were effectively concentrated in the recovery solution after the MNRC was operated for a whole concentrating test of 120 h, consisting of five operational cycles (Fig. 3A). The concentrations of NH_4_^+^-N in the recovery solution increased from 0 to 35.7 mg/L and 37.8 mg/L at the end of the first and second concentrating tests respectively. As the initial concentration of NH_4_^+^-N in synthetic wastewater was 23.8 mg/L, these results indicate that NH_4_^+^-N could be driven into the recovery solution and concentrated to 1.5 times. Meanwhile, PO_4_^3−^-P concentration in the recovery solution reached 16.0 and 14.2 mg/L at the end of the first and the second concentrating tests, which were 2.5 and 2.2 times the initial concentration in the synthetic wastewater (6.4 mg/L). COD concentrations in recovery solutions were measured as less than detection limit, which confirms that the ion exchange membranes blocked the most of organics in the electrode chambers from polluting the recovery solution. These results demonstrate that the MNRC could collect and concentrate NH_4_^+^-N and PO_4_^3−^-P simultaneously in the recovery solution without COD contamination detected.

The slopes of the concentration curves for NH_4_^+^-N and PO_4_^3−^-P decreased during the concentrating test, indicating a slowing of ion recovery. The recovery process was driven by electrical power and obstructed by the diffusion of ions from the recovery chamber to the electrode chambers (opposite migration direction to the recovery process). The current output performance was repeatable in each operational cycle, demonstrating that the driving force remained relatively consistent during the concentrating tests (Fig. 3C). When fed with fresh synthetic wastewater with sufficient substrate (COD) and neutral pH, the MNRC could generate a reproducible maximum current of ~0.4 mA (0.56 A/m^2^). The stable COD removal efficiency for each operational cycle was consistent with repeatable current producing performance (Fig. 3B). The pH changes of the electrolyte were also repeatable for each operational cycle, decreasing from 6.8 to ~4.0 (Supporting Information, Fig. S1). As the current generation was repeatable, diffusion is likely to be the main factor leading to the deceleration of nutrient recovery. When nutrient ions accumulated in the recovery chamber, the diffusion of ions driven by increased concentration gradients across membranes partially offset the recovery process. Other kinds of ions including Na^+^, H^+^, Cl^−^ and SO_4_^2−^ kept migrating from electrode chambers to the recovery chamber and supplemented the role of nutrient ions to conduct internal current (data and analysis was shown in the following section). Maximum concentration was achieved when the driving force of the electrical field equaled that of the concentration gradients of nutrient ions across the membranes. To achieve higher nutrient concentration in the recovery chamber, the MNRC current should be promoted, for example by using wastewater containing higher COD, enlarging the reactor or improving the structure of the reactor, such as using thinner chambers to reduce the solution resistance.

Removal efficiencies for COD, NH_4_^+^-N, and PO_4_^3−^-P were >82%, >96%, and >64%, respectively, in all the 10 operational cycles during the two concentrating tests (Fig. 3B). This indicates that the continuous recovery of NH_4_^+^-Ns and PO_4_^3−^-Ps in the recovery solution facilitated steady removal from the synthetic wastewater. The average total removal quantity of PO_4_^3−^-P in the two concentrating tests was 1.9 ± 0.1 mg, with total recovery of 1.2 ± 0.1 mg. This result shows that approximately 63% of the removed PO_4_^3−^-P was recovered in the recovery solution. The removal performance of NH_4_^+^-N remained high throughout the concentrating tests (Fig. 3B). The average total removal and recovery quantities of NH_4_^+^-N in the two concentrating tests were 11.6 ± 0.1 mg and 2.8 ± 0.1 mg. This indicates that the NH_4_^+^-N that diffused into the recovery solution, driven by the electrical field, only accounted for 24% of the total removed quantity, whereas more than three quarters of the NH_4_^+^-N was removed by other paths. Anion-exchange chromatography showed that concentrations of NO_3_^−^ and NO_2_^−^ were below detectable limits, and therefore the presence of these ions could not be confirmed. The result of abiotic control shows that, the concentrations of NH_4_^+^-N and PO_4_^3−^-P in the wastewater effluent were nearly the same as that in the initial wastewater (Supporting Information, Fig. S2). In contrast, the result of open circuit control experiment shows that, even without current production, about 60% of NH_4_^+^-N and 16% of PO_4_^3−^-P were removed. It was probably via bio-uptake of microorganisms in the electrode chambers and circulation tubes of MNRC, physical adsorption, chemical precipitation, or membrane fouling, etc. In addition, the nitrification and denitrification might take place in the cathode chamber and thus result in the loss of the nitrogen. The overall mass balances and mechanisms in nutrient removal will be further investigated in the future to illustrate the path to improve the performance of this system.

### Component changes and charge transfer in the MNRC

**Figure 4:**
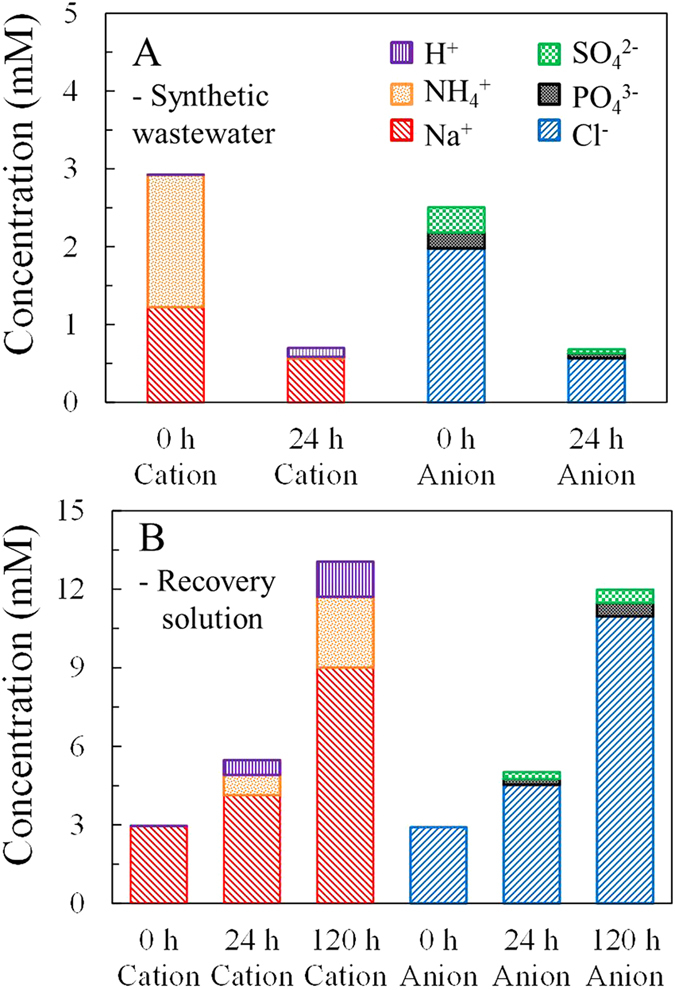
Ion distributions in wastewater at the beginning and end of the first operational cycle during a concentrating test (A), and ion distributions in the recovery solution at the beginning and end of the first operational cycle, and the end of the concentrating test (B).

The concentrations of all types of ions, except H^+^, in the synthetic wastewater were reduced after the MNRC completed one operational cycle (Fig. 4A). Cations in the wastewater at 0 h included 1.70 mM NH_4_^+^, 1.22 mM Na^+^, and 2 × 10^−4^ mM H^+^ (according to the pH, 6.8). After 24 h operation, 1.67 mM NH_4_^+^ and 0.66 mM Na^+^ were removed, while 0.1 mM H^+^ was gained to balance electric charge. Anion tests showed 1.97 mM Cl^−^, 0.20 mM orthophosphate (referred to as PO_4_^3−^), and 0.32 mM SO_4_^2−^ in the initial wastewater, with 0.57 mM (Cl^−^), 0.06 mM (PO_4_^3−^), and 0.05 mM (SO_4_^2−^) remaining after 24 h. At both the beginning and end of an operational cycle, the total electric charges of cations equaled those of anions in the synthetic wastewater. These results indicate that the MNRC could remove NH_4_^+^, PO_4_^3−^, and other ions from the wastewater. In addition to the removal of NH_4_^+^ (98%) and PO_4_^3^ (70%) discussed above, 54% of Na^+^, 71% of Cl^−^, and 84% of SO_4_^2−^ were also removed from the synthetic wastewater.

Concentrations of all types of ions in the recovery solution kept increasing throughout the 120 h concentrating test (Fig. 4B). After the initial 24 h (i.e., at the end of the first operational cycle), the concentrations of cations increased by 1.18 mM for Na^+^, 0.78 mM for NH_4_^+^, and 0.74 mM for H^+^, whereas those of anions increased by 1.63 mM for Cl^−^, 0.21 mM for PO_4_^3−^, and 0.27 mM for SO_4_^2−^ in the recovery solution. These results showed that the migration of Na^+^ was quicker than that of NH_4_^+^, and the competitive migration order of anions was Cl^−^ > PO_4_^3−^ > SO_4_^2−^ under the conditions used in this experiment. H^+^ was considered to balance the electrical charge, and its concentration changes are not discussed further here. After 120 h the total recovered quantities of those ions were 6.04 mM Na^+^, 2.70 mM NH_4_^+^, 1.35 mM H^+^, 8.06 mM Cl^−^, 0.52 mM PO_4_^3−^, and 0.49 mM SO_4_^2−^ in the recovery solution. The increments of Na^+^ and Cl^−^ at 120 h were 5.1 and 4.9 times those in the first 24 h, demonstrating that these two ions maintained a steady rate of migration from synthetic wastewater to the recovery solution throughout the concentrating test. However, the final concentrations of NH_4_^+^, PO_4_^3−^, and SO_4_^2−^ were 3.5, 2.5, and 1.9 times those in the first operational cycle, which indicates that the migration rates of those ions decreased in the latter part of the concentrating test. The molar conductivity, radius, mobility, and concentration of each ion, in combination with the electrical field intensity and exchange selectivity of membrane, were factors affecting migration processes in the solutions and across the membranes^32^,^33^. Further studies will be conducted to better understand the competitive migration characteristics. It should be mentioned that ion recovery quantities were less than removal quantities for all types of ions examined in the MNRC, with losses of: 11% Na^+^, 77% NH_4_^+^, 42% Cl^−^, 27% PO_4_^3−^, and 51% SO_4_^2−^. Those ion losses might be due to bio-uptake, adsorption, precipitation, membrane fouling, or measurement error, etc. The mechanisms of the ion losses will be further investigated in the future.

### Charge demands analysis of ions in the MNRC

**Figure 5:**
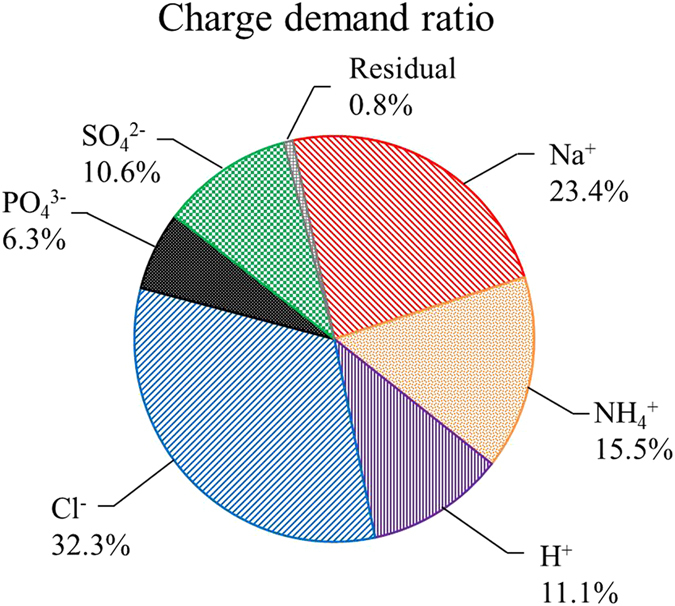
Charge demand ratio of each type of ion in the recovery solution during a 24 h operational cycle.

Different kinds of ions along with NH_4_^+^-N and PO_4_^3−^-P in the MNRC were transferred through ion exchange membranes (Fig. 5). Total charge demand ratios were 50% for cations and 49.2% for anions, indicating that the charge transfer efficiency of the MNRC was 99.2%. The migration of NH_4_^+^ required 15.5% of the total charge, which was less than that of Na^+^ (23.4 %) and higher than that of H^+^ (11.1%). Meanwhile, the charge demand ratio of PO_4_^3−^ was 6.3%, which was the lowest compared with those of 32.3% for Cl^−^ and 10.6% for SO_4_^2−^. Na^+^ and Cl^−^ each occupied the highest electrical transfer capacity of the MNRC among all the cations and anions. The results are attributed to the combined effects of ionic characteristics (such as size, charge and concentration), electrical field, and membrane exchange selectivity, as discussed in the previous section. Thus, the competition between desired ions (NH_4_^+^ and PO_4_^3−^) and other ions will be further investigated to obtain higher recovery performance. Additionally, the transfer of H^+^, which was used to balance solution pH, also required remarkable electrical charges, and thus effective methods of stabilizing acidity might promote the collection of NH_4_^+^.

## Discussion

The MNRC developed in this study is a proof-of-concept that might enable simultaneous wastewater purification and nutrient recovery without additional energy input. Concentrations of COD, NH_4_^+^-N and PO_4_^3−^-P in the effluent of MNRC were effectively reduced compared to those in the influent, while NH_4_^+^-N, and PO_4_^3−^-P were concentrated into the recovery solution. Concentrated nutrient ions in the recovery solution could benefit further recovery of NH_4_^+^-N, and PO_4_^3−^ -P (for example struvite production)^6^,^34^. After the nutrients are recovered, the remaining recovery solution which mainly contains NaCl could be reused in the MNRCs to concentrate NH_4_^+^ and PO_4_^3−^ in subsequent recovery processes. Thus the recovery solution can be recycled to continuously recover nutrients from wastewater. It was reported that an MFC combined with struvite formation in the anode effluent could produce struvite based on the nutrient ions in wastewater when specific types of wastewater with higher nutrient concentration, such as urine or swine wastewater, were applied^35^,^36^. The struvite formation was determined by the concentrations of magnesium, ammonium and phosphate^5^. Therefore higher concentrations of nutrient ions could promote the struvite production and save magnesium salt addition. Different from the previous reports, the MNRC could obtain a recovery solution with concentrated ammonium and phosphate compared to those in the initial wastewater thus the objective wastewater type might be expanded to municipal wastewater.

The internal resistance of a BES is relatively high when treating the municipal wastewater with a low conductivity. In the configuration of MNRC, the anode chamber was filled with the granular activated carbon and the cathode chamber was made as thin as possible to minimize the internal resistance. In a BES, buffer solution was always used to improve the conductivity and balance the pH of the electrolyte, while the consumption of buffer solution might increase the operating cost in the practical application. To avoid the addition of buffer, the recirculation mode was chosen to achieve the acid-base neutralization using the H^+^ and OH^−^ generated by the electrode reactions themselves, which was attempted in a previous work^37^. Based on the above approaches, the MNRC system could run efficiently in the low conductivity condition without buffer solution.

The real wastewater contains the charged organic substances. In some previously reported wastewater treatment studies based on the BES system, charged organic molecules can also migrate across the ion exchange membranes^38^,^39^. Considering the practical application of this system, an abiotic MNRC construction was operated under an applied current of 5 mA to treat the real wastewater sampled from a local sewage treatment plant (Gaobeidian, Beijing, China). The concentration of COD in the recovery solution was measured as ~1 mg/L after this experiment. This result indicates that the charged organic fractions (humic acid etc.) in the real domestic wastewater could migrate into the recovery solution but in a small amount with the experimental condition of the MNRC system. The large molecular size and weight of the charged organic molecules might be the reason for this result. Besides, at relatively low pH of the wastewater and recovery solution (Supporting Information, Fig. S1), the primary species of humic acid and bicarbonate are in molecular forms with neutral charge^40^, thus their migration competing with nutrient ions might be relatively weak. Further research on real wastewater is needed to investigate the dynamic balance between concentrated NH_4_^+^ and PO_4_^3−^ and other types of ions in the recovery solution during the long-term operation.

The proof-of-concept MNRC has achieved nutrient recovery using the energy contained in the wastewater. However, the concentrations of NH_4_^+^ and PO_4_^3−^ achieved in the recovery solution were not high enough. Since the current is the driving force of all ions’ migration, the current generated by the MNRC should be promoted to enhance the concentrating and removal of the nutrients. Approaches for increasing the current production include enlarging the scale of the reactor, optimizing the configuration (such as using thinner chambers) and optimizing operation conditions (solution flow rate, etc). Besides, when the objective wastewater contained high concentrations of nutrient ions (for example diluted human urine) and COD, the current output of MNRC was expected to increase and the rate and extent of recovery might be enhanced^30^. In addition, the volume ratio of the electrolyte and the recovery solution may affect the final N and P concentrations in the recovery solution. This ratio was 2:1 in the present study. When this ratio enlarges, the concentrating results might be enhanced as the nutrient ions could accumulate in a smaller volume of recovery solution. A previous study on a stacked MDC has proved that the smaller volume of solution could accumulate higher concentration of salt^41^. The effect of the volume ratio on the nutrient removal and recovery performance will be further investigated in the future.

The coulombic efficiencies were relatively low (ranging from 7% to 15%), indicating that the chemical energy contained in wastewater was not fully utilized in the process of producing electricity. It was probably caused by the contact of wastewater with the oxygen in the cathode chamber and the COD consumption of microorganisms which did not participate in the current production. The result of open circuit control experiment demonstrates that without current production, about 66% of COD was consumed by the metabolism of microorganisms (both in the electrode chambers and the tube system) (Supporting Information, Fig. S2). To enhance the coulombic efficiency, future studies will seek to optimize the MNRC configuration and operation mode. The ion transfer process also needs to be researched to reveal the competitiveness of NH_4_^+^-N and PO_4_^3−^-P with other kinds of ions, to make better use of the electric power by the MNRC and thus produce useful products.

## Methods

### MNRC construction

Each chamber of an MNRC was a Plexiglas cube, with an inner diameter of 3.0 cm. The widths of these three chambers were 3 cm (anode), 0.5 cm (recovery), and 0.5 cm (cathode), and the effective volumes of these chambers were 21.2 mL (anode), 3.6 mL (recovery), and 3.6 mL (cathode). The anode chamber and the recovery chamber were separated by a cation exchange membrane (CEM, Ultrex CMI7000, Membrane International Inc.). The recovery chamber and the cathode chamber were separated by an anion exchange membrane (AEM, Ultrex AMI-7001, Membrane International Inc.). The anode chamber was filled with granular activated carbon (~1 mM in diameter, ~2 to 5 mM in length, Beijing Chunqiudingsheng Environmental Science and Technology Co. Ltd., China)^42^ to hold electrically active biofilms and serve as an anode of the MNRC. A titanium mesh was added in the anode chamber, against the CEM, to serve as an anode current collector. The air cathode was made of carbon cloth (30% wet-proofing) with 0.5 mg/cm2 platinum catalyst and four polytetrafluoroethylene (PTFE) diffusion layers^43^. A glass fiber separator (1.0 mM thickness, DC1.0, Jiafu Co., China) was placed against the air cathode^44^,^45^. The synthetic wastewater was recirculated from a 100 mL container to the anode chamber, then to the cathode chamber, and back to the container. The tube connecting the anode and cathode chambers was 30 cm in length, as in a previous report^37^. The recovery solution was continuously circulated between a 50 mL container and the recovery chamber, via a tube ~50 cm in length. The circulation flow rates of electrolyte and recovery solution were kept the same in all experiments.

### Microorganisms and medium

The granular activated carbon used in the anode chamber was inoculated from an MFC operated for 6 months. The electrolyte was synthetic wastewater that imitated typical domestic wastewater, containing (per liter in deionized water): 0.4 g glucose, 0.020 g NaH_2_PO_4_·2H_2_O, 0.021 g Na_2_HPO_4_·12H_2_O, 0.089 g NH_4_Cl, 0.016 g NaCl, 0.041 g Na_2_SO_4_, and 12.5 mL of trace mineral metals solution^46^. All experiments were conducted using the same synthetic wastewater solution with measured COD concentration of 369 ± 21 mg/L, measured NH_4_^+^-N concentration of 23.8 ± 1.3 mg/L, and measured PO_4_^3−^-P concentration of 6.4 ± 0.6 mg/L. The draw solution was 0.164 g/L NaCl solution in most experiments except as noted.

### Experimental procedures

The MNRC anodes were acclimated under MFC mode (no recovery chamber). A bottle of 100 mL synthetic wastewater was recirculated at a flow rate of 15 mL/min between the anode and cathode chambers of the MFCs, and was renewed every 24 h, which was defined as an operational cycle. After all the MFCs exhibited stable and parallel performance, with repeatable maximum voltage of ~600 mV (1000 Ω) for over 10 cycles, these MFC reactors were transformed to the MNRC configuration. The MNRCs were operated with 100 mL synthetic wastewater as electrolyte and 50 mL 0.164 g/L NaCl solution as recovery solution under the same flow rate of 15 mL/min with electrolyte, driven by a peristaltic pump (BT100-1 L, Lange, China). The external resistance of the MNRC was gradually decreased from 1000 Ω to 5 Ω to achieve the peak current, with each resistance used for three full cycles^47^.

When these MNRC reactors could steadily produce current, a preliminary experiment was conducted to optimize flow rates. The MNRCs were operated at flow rates of 5, 15, and 25 mL/min and achieved the highest current production at 15 mL/min (Supporting Information, Fig. S3). Based on this result, subsequent experiments all used the optimal flow rate of 15 mL/min. To investigate the mass balance of NH_4_^+^-N and PO_4_^3−^-P, an abiotic control (using the MNRC reactor without anode inoculation to operate for 24 h) and an open circuit control (using the working MNRC to operate in open circuit for 24 h) experiments were conducted with the flow rate of 15 mL/min.

As the NaCl contained in recovery solution was used to facilitate internal current conduction, the influence of initial NaCl concentrations on MNRC performance was also investigated. The recovery chamber was supplied with 0, 164, and 328 mg/L NaCl solutions, respectively, to optimize the concentration of recovery solution. The optimal concentration of NaCl solution of 164 mg/L was used for the rest of the experiments.

To investigate the recovery performance and concentrating extent of ammonium and phosphate, concentrating tests were conducted in which recovery solution was circulated but not replaced during several operational cycles. The pH as well as concentrations of COD, NH_4_^+^-N, and PO_4_^3−^-P in both synthetic wastewater and recovery solution were measured at the end of each operational cycle (24 h). The concentrating test was completed after 120 h, when the concentration of ammonium or phosphate in the recovery solution approached peak level. The concentrating tests were repeated and all experiments were conducted in duplicate reactors at room temperature (~25 °C).

### Analyses and calculations

A data acquisition system (2700, Keithley Instrument, OH, USA) was used to monitor the output voltage (*U*) of MFCs and MNRCs every 20 min throughout the operation period. According to Ohm’s law, the MNRC current (*I*) (mA) was calculated as *I* = *U*/*R*, in which *R* was external resistance (Ω).

Concentrations of COD, NH_4_^+^-N, and PO_4_^3−^-P were determined by standard methods^48^. Concentrations of SO_4_^2−^ and Cl^−^ were measured using ion chromatography (ICS-1100, DIONEX, USA). Na^+^ concentration was measured using inductively coupled plasma atomic emission spectrometry (ICP-AES, IRIS Intrepid II XSP, Thermo, USA). The pH was monitored by a pH meter (Inlab 731, Mettler Toledo, USA).

Theoretical charge demand for a certain ion’s migration was defined as the electric charge that was theoretically required for those ions to migrate from electrode chambers to recovery chamber. The total theoretical charge demand was the sum of the theoretical charge demands for all kinds of ions. The total actual charge demand was the total charges passed through the external circuit of the MNRC over an operational cycle, which was calculated as 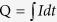, in which *I* was the output current. The ratio of total theoretical charge demand to total actual charge demand was defined as charge transfer efficiency. The ratio of each ion’s theoretical charge demand was defined as the charge demand ratio of the MNRC, calculated by:


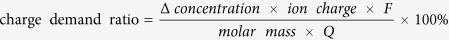


in which Δ *concentration* was the concentration increment of a certain kind of ion in the recovery solution; F is Faraday’s constant, 96485 C/mol. In the case of PO_4_^3−^-P, the average electrical charge of ions was larger than 1 and smaller than 2, since the main ionic forms of phosphate were H_2_PO_4_^−^ (>80%) and HPO_4_^2−^ (<20%) in the solutions in this study. Thus, 1.2 was assumed as the average electric charge of PO_4_^3−^-P ions for calculation and estimation, although this might introduce some errors.

## Additional Information

**How to cite this article**: Chen, X. *et al.* Novel Self-driven Microbial Nutrient Recovery Cell with Simultaneous Wastewater Purification. *Sci. Rep.* **5**, 15744; doi: 10.1038/srep15744 (2015).

## Supplementary Material

Supplementary Information
