# Supplementary material for: Novel Self-driven Microbial Nutrient Recovery Cell with Simultaneous Wastewater Purification
Source: Sci Rep. 2015 Oct 27;5:15744. doi: 10.1038/srep15744 (PMC4621542; doi:10.1038/srep15744)
Supplement: Supplementary Information [file srep15744-s1.doc]

**Supplementary information**

**Novel Self-driven Microbial Nutrient Recovery Cell with Simultaneous Wastewater Purification**

Xi Chen, Dongya Sun, Xiaoyuan Zhang^*^, Peng Liang, Xia Huang^*^

State Key Joint Laboratory of Environment Simulation and Pollution Control,

School of Environment, Tsinghua University, Beijing 100084, P.R.China

* Corresponding author: E-mail: xhuang@tsinghua.edu.cn;

phone: (86)10-62772324; fax: (86)10-62771472

* Co-corresponding author: E-mail: zhangxiaoyuan@tsinghua.edu.cn

Number of pages: 2

Number of figures: 3

**Figure S1** pH changes in wastewater and recovery solution during two repeated 120 h concentrating tests.

**Figure S2** Concentrations of COD, NH_4_^+^-N and PO_4_^3-^-P in the initial wastewater and in the effluents after abiotic, open circuit and closed circuit tests over a 24 h operational cycle. The recovery solution contained 164 mg/L NaCl. Abiotic control test: using the MNRC reactor without anode inoculation to operate; Open circuit test: using the working MNRC to operate in open circuit; Closed circuit test: using the working MNRC to operate with the external resistance of 5 Ω.

**Figure S3** Current generation of the MNRC under different flow rates.
